# Irradiated Non-replicative Lactic Acid Bacteria Preserve Metabolic Activity While Exhibiting Diverse Immune Modulation

**DOI:** 10.3389/fvets.2022.859124

**Published:** 2022-05-18

**Authors:** Luca Porfiri, Johanna Burtscher, Richard T. Kangethe, Doris Verhovsek, Giovanni Cattoli, Konrad J. Domig, Viskam Wijewardana

**Affiliations:** ^1^Animal Production and Health Section, Joint Food and Agriculture Organization (FAO)/International Atomic Energy Agency (IAEA) Centre of Nuclear Techniques in Food and Agriculture, International Atomic Energy Agency, Vienna, Austria; ^2^Department of Food Science and Technology, Institute of Food Science, University of Natural Resources and Life Sciences, Vienna, Austria; ^3^VetFarm Medau, University of Veterinary Medicine Vienna, Berndorf, Austria

**Keywords:** lactic acid bacteria, gamma irradiation, vaccine adjuvant, immune modulation, metabolic activity

## Abstract

In the recent years, safety concerns regarding the administration of probiotics led to an increased interest in developing inactivated probiotics, also called “paraprobiotics”. Gamma irradiation represents a promising tool that can be used to produce safe paraprobiotics by inhibiting replication while preserving the structure, the metabolic activity, and the immunogenicity of bacteria. In this study, we evaluated the ability of four strains of lactic acid bacteria (LAB: *Lacticaseibacillus casei, Lactobacillus acidophilus, Lactiplantibacillus plantarum*, and *Lacticaseibacillus paracasei*) in preserving the metabolic activity and the immune modulation of swine porcine peripheral blood mononuclear cells, after gamma irradiation or heat inactivation. Our results show that all four strains retained the metabolic activity following gamma irradiation but not after heat inactivation. In terms of immune-modulatory capacity, irradiated *L. acidophilus* and *Lc. paracasei* were able to maintain an overall gene expression pattern similar to their live state, as heat inactivation did with *Lc. casei*. Moreover, we show that the two inactivation methods applied to the same strain can induce an opposed expression of key genes involved in pro-inflammatory response (e.g., IFNα and interleukin-6 for *Lc. casei*), whereas gamma irradiation of *L. acidophilus* and *Lc. paracasei* was able to induce a downregulation of the anti-inflammatory TGFβ. Taken together, our data show that immune modulation can be impacted not only by different inactivation methods but also by the strain of LAB selected. This study highlights that gamma irradiation harbors the potential to produce safe non-replicative metabolically active LAB and identifies immunomodulatory capacities that may be applied as vaccine adjuvants.

## Introduction

Most probiotics belong to the group of gram-positive, non-pathogenic lactic acid bacteria (LAB) (1), which can be found in different niches (e.g., plants, milk, and gastrointestinal tracts) (2) and are able to produce large amounts of lactic acid by fermenting carbohydrates, often linked to health-promoting effects (3). Bifidobacteria and lactobacilli are among the most extensively studied probiotic LAB (4, 5). The generic term “lactobacilli” refers to all genera that were classified as *Lactobacillaceae* until a reclassification and introduction of 25 new genera in 2020 (6). Specifically, some lactobacilli strains, such as the ones evaluated in this study, *Lacticaseibacillus casei, Lactobacillus acidophilus, Lactiplantibacillus plantarum*, and *Lacticaseibacillus paracasei*, have been proven to be particularly capable of stimulating both the intestinal mucosa and the systemic immune response, thus taking the name of “immunobiotics” (7), showing both Th1 and Th2 responses (8–10), which undoubtedly represent remarkable intrinsic adjuvanticity capacities (11, 12). These immunomodulatory effects are exerted through their interaction with different types of immune cells, including lymphocytes, NK cells, and antigen-presenting cells (5, 7). What is intriguing is the capacity of some LAB to induce a balanced pro- and anti-inflammatory action (13). This is achieved by the activation of different pathways leading to a broad and diverse expression of T helper cell subsets, such as Th1, Th2, Th17, and T-regulatory (Treg), inducing the production of different sets of cytokines (14). A broad and diverse immune stimulation is a common characteristic of vaccine adjuvants (15), and some LAB strains have displayed a notable potential for their application in several vaccine formulations (16–18).

Nevertheless, there is an aspect that needs to be considered and evaluated regarding the administration of probiotics, and this regards safety; in fact, although many lactic acid bacteria strains are considered as Generally Recognized as Safe (GRAS), in the recent years, several potential side effects have been documented for some strains, including intestinal probiotic overgrowth, gastrointestinal symptoms (e.g., vomiting, diarrhea, and nausea), bloodstream infection (e.g., bacteremia, sepsis, and peritonitis), excess D-lactate production, dysbiosis, and horizontal gene transfer (19–21). The latter is of particular concern because it can severely contribute to the diffusion of antibiotic resistance, already representing a global threat to human and animal health (22). Considering these factors, current literature review is fostering a debate on whether probiotics need exclusively to be “alive” to induce health benefits to the host organism (23). In fact, more evidence is emerging that also, nonviable probiotic strains are able to provide beneficial effects (24). Since most of the live probiotics ingested are not able to survive the harsh condition of the stomach and intestine, resulting in a severely affected viability of these products (25), most of the health benefits related to probiotics may be attributable to their metabolites and their cell surface components (24), which would therefore be independent from whether they are administered live or inactivated. This noteworthy perspective led to a new interest to explore the application of the so-called paraprobiotics (also called “ghost,” or inactivated probiotics) (26), which in addition to being safer (27), which would have advantages in terms of longer shelf life and more favorable storage and transport conditions (24), especially to those areas where strict handling conditions cannot be met (e.g., developing countries) (23).

Among the different methods of inactivation to generate paraprobiotics (26, 28), gamma irradiation technology, which is usually applied to inactivate or sterilize microbes (29), can be used, at optimal doses, to stop the replication of bacteria and parasites while preserving their structure and their metabolic activity (30). This leads to inactivated bacteria which is defined as “metabolically-active non-replicative,” potentially preserving all (or most of) the characteristics of the live bacterium while guaranteeing a high level of safety. Evidence showed that gamma irradiation successfully protected surface antigens and cell composition of bacteria compared to other means of inactivation (e.g., heat treatment) (31). Despite the great potential of this technology, few studies in the literature explored the application of gamma irradiation to generate paraprobiotics. Almada et al. (28), for instance, have explored how different strains of lactobacilli (and bifidobacteria) display a different degree of resistance to gamma irradiation, besides investigating several other aspects, such as cultivability, integrity, and physiology. Raz and Rachmilewitz (32) indicated how a mix of paraprobiotics (among which *Lc. casei, Lp. plantarum*, and *L. acidophilus*), obtained by radiation, was more effective in the treatment of colitis in animal models than those inactivated by heat treatment. In contrast, according to Kamiya et al. (33), neither gamma irradiation nor heat inactivation was effective in preserving the inhibitory capacity on visceral pain induced by colorectal distension in rats of *L. reuteri*.

Therefore, in this comparative study, we investigated the ability of four different LAB strains, irradiated with gamma rays, inactivated with heat treatment, and in their live form, to stimulate the immune response of *ex vivo* porcine peripheral blood mononuclear cells (PBMCs), by evaluating the gene expression of 26 immune markers (related to transcription factors, pathogen recognition receptors, innate and adaptive immune response) using quantitative real-time PCR. The application of this panel aims at analyzing the expression of different immune markers involved in different pathways and immunological responses at the same time, providing a broader picture and a more extensive understanding, compared to similar studies, of the immune modulation exerted by this type of lactic acid bacteria.

## Materials and Methods

### Preparation of Bacterial Suspensions

A total of four strains of LAB were used for the experiments in this study: *L. acidophilus* (*L. acidophilus* LMG 9433, type strain), *Lp. plantarum* subsp. *plantarum* (*Lp. plantarum* DSM 20205), *Lc. casei* (*Lc. casei* LMG 6904, type strain), and *Lc. paracasei* subsp. *paracasei* (*Lc. paracasei* LMG 12586). All cultivation steps were performed in Lactobacillus broth acc. to De Man, Rogosa and Sharp (MRS) broth (Merck KGaA, Darmstadt, Germany) under anaerobic conditions using a jar gassing system (gas mixture containing 80% N_2_, 10% CO_2_, and 10% H_2_; Don Whitley Scientific, West Yorkshire, UK) at 37°C (LMG 9433) or 30°C (DSM 20205, LMG 6904, and LMG 12586). Cell suspensions for inactivation experiments were produced by transferring 800 μl of an overnight culture of each strain into 80 ml of pre-warmed MRS broth followed by an anaerobic incubation for 24 h. MRS broth was removed by centrifugation at 8,000 × *g* for 6 min and by discarding the supernatant. Bacterial biomass was washed by two cycles of resuspension of the biomass in 40 ml of sterile phosphate-buffered saline (PBS, Merck KGaA, Darmstadt, Germany) and subsequent centrifugation as described above. The resulting supernatants were discarded. Subsequently, based on OD measurements at 625 nm, the samples were diluted with PBS to reach the target concentration of 10^8^ cfu/ml. The suspension was centrifuged again using the conditions described above, and the resulting biomass without supernatant was resuspended in 75% v/v of the original volume of PBS. The remaining 25% v/v was supplemented with 1 M trehalose, resulting in approximately 80 ml of bacterial suspension containing 10^8^ cfu/ml. Aliquots (2.3 ml each) of the final suspension were distributed into 32 cryovials and stored at −80°C until further analysis.

### Gamma Irradiation and Heat Inactivation of LAB Strains

Gamma irradiation was performed at International Atomic Energy Agency (IAEA) laboratories in Seibersdorf (Austria) using Cobalt60-source 812 Irradiator from Foss Therapy Services, Inc (34). About eight doses were used to assess the D10 dose: 250, 500, 750, 1,000, 1,500, 2,000, 2,500, and 3,000 Gy. About 50-ml Falcon conical tubes, containing the cryovials, were placed inside a 2.5 l Bio-Bottle (Orange Bio-Bottle; UN Specification Mark: 4GU/Class 6.2) filled with dry ice, to maintain the frozen state, which was eventually inserted into the irradiator. Following irradiation, vials were kept at −80°C until decimal dilutions of all samples. Controls were streaked on MRS agar (Merck KGaA, Darmstadt, Germany) and incubated anaerobically for 72 h to determine viable cell counts in cfu/ml. Next, the survival fraction percentage of each strain of LAB was determined against the different irradiation doses tested to calculate the D10 value.

D10 value is defined as the ability of gamma irradiation to reduce an exposed microbial population by 90%(one log_10_) under standard conditions of time, temperature, and dose. This value for the different LAB strains was calculated using the inverse of the slope of the regression lines (−1/slope) of gamma irradiation dose against survival fraction (log) (35, 36) using GraphPad Prism version 9.1.2 for (GraphPad Software, San Diego, California USA, www.graphpad.com). Once the D10 value was assessed, the minimum dose needed for the complete inactivation of bacteria was determined by multiplying the D10 value × log concentration of the batch. To have a safety margin and for the easiness of delivering a precise gamma irradiation dose over multiple experiments, we added 1.5 of D10 dose on top of the estimated lethal dose and rounded up to the nearest 500 Gy. These additional irradiation doses provided were herein being termed as “safety inhibitory dose (SID).”

A total of three doses of gamma irradiation were used to assess metabolic activity: a low universal dose to reduce growth (3,000 Gy), a strain-dependent SID, and a high universal dose (10,000 Gy). The procedure described above was then applied to irradiate LAB at these three doses. Additional samples were prepared (and aliquoted) as described above, inactivated *via* heat treatment at 95°C for 10 min, and finally stored at −80°C. Colony counts were done as described above following irradiation at 3,000 Gy, SID, 10,000 Gy, or after heat treatment.

### Metabolic Activity and Membrane Integrity

Metabolic activity of live, gamma-irradiated, and heat-inactivated bacteria was determined by measuring redox potential (using the resazurin-based cell-permeable compound Alamar blue) and by adenosine triphosphate (ATP) production. The Alamar blue assay was performed using Alamar blue cell viability reagent (Thermo Fisher: catalog no. DAL1025) according to the manufacturer's protocol. Briefly, frozen LAB samples were thawed and mixed well, and 90 μl bacterial suspension was added to black 96-well assay plates. These plates were incubated at 37°C for 15 min, and then, 10 μl of Alamar blue solution was added and was incubated for another 2 h at 37°C. The metabolic activity was measured as the fluorescence intensity emitted at 590 nm (excitation at 560 nm) using a microplate reader. ATP production was measured with the BacTiter-GloTM Microbial Cell Viability Assay (Promega, Madison, WI, United States) according to the manufacturer's protocol. Briefly, 100 μl of LAB samples was added to opaque-walled 96-well plates and were incubated at 37°C for 15 min. Next, 100 μl of BacTiter-Glo™ Reagent was added, mixed, and then incubated at room temperature for 5 min in a shaker. Following the incubation, the luminescence was measured using a microplate reader. ATP concentrations were calculated from a standard curve.

Membrane integrity was measured using LIVE/DEAD™ BacLight™ Bacterial Viability Kit (Molecular Probes®, Grand Island, NY, United States). This kit contains mixtures of SYTO® 9 green-fluorescent nucleic acid stain and the red-fluorescent nucleic acid stain, propidium iodide. The SYTO 9 stain labels all bacteria in a population while propidium iodide penetrates only bacteria with damaged membranes. The assay was performed according to the manufacturer's protocol with slight modifications. Briefly, 100 μl of 10-fold diluted samples was aliquoted into dark 96-well assay plates, and 1 μl of propidium iodide and SYTO 9 mixture was added and mixed. After 15 min of incubation at 37°C, plates were read at 485/530 (excitation/ emission) and 485/630 (excitation/ emission). The membrane integrity was assessed as fluorescence intensity of SYTO 9/propidium iodide.

### Isolation of Swine PBMCs and Their Stimulation

Blood samples were obtained from healthy adult sows, aged between 12 and 48 months, raised in the teaching and research farm of the University of Veterinary Medicine, Vienna, Austria. Whole blood was obtained by puncture of the jugular vein with heparinized Primavette® V Li.-Heparin 10-ml tubes (Kabe Labortechnik GmbH, Germany). Blood collection and animal handling were performed according to the accepted animal welfare standards (37). None of the animals included in the study showed any signs of clinical disease. The herd is free of Porcine Reproductive and Respiratory Syndrome Virus (PRRSV) and the sows were vaccinated against porcine parvovirus (PPV), porcine circovirus type 2 (PCV-2), and *Erysipelothrix rhusiopathiae*. The blood collection was approved by the University of Veterinary Medicine Vienna's Ethics and Animal Welfare Committee and the Austrian Ministry of Research and Science's Advisory Committee for Animal Experiments (BMBWF-68.205/0192-V3b/2018). Swine PBMCs were isolated and handled as described previously for other species (38). Briefly, fresh blood was first carefully layered over Ficoll-Paque PLUS (Sigma) in a 50-ml Falcon conical tube. Successively, PBMCs were isolated by density gradient centrifugation for 35 min at 800 × *g* at 20°C, allowing the collection at the plasma/Ficoll interface using a Pasteur pipette, and washed three times with PBS to remove the platelets and cell debris. PBMCs were then resuspended in complete medium containing RPMI 1640, 10% fetal bovine serum (FBS), and a solution containing penicillin, streptomycin, and amphotericin B (Antibiotic-Antimycotic, Thermo Fisher Scientific) at a concentration of 10 × 10^6^ cells/ml. Following, cells were incubated at 37°C in an atmosphere of 5% CO_2_ for 2 h to remove any contaminating bacteria or molds. Next, antibiotics and antimycotic were removed with two washing cycles using only medium (RPMI 1640), centrifuging at 1,500 rpm at 4°C for 7 min. The resulting cell pellet was resuspended using antibiotic-free medium (RPMI 1640, 10% FBS) at a concentration of 4 × 10^6^ cells/ml, and aliquots of 5 ml per well were distributed in 6-well plates. Next, PBMCs were incubated without (negative control) or with 50 μl of either live, gamma-irradiated, heat-inactivated LAB or various stimulation cocktails as positive controls (concanavalin A or phorbol 12-myristate13-acetate and ionomycin or pokeweed mitogen) at a concentration described previously (38). In each single experiment, PBMCs from one animal were stimulated with all the strains selected (and with various treatments), and the procedure was replicated for each of the five animals. During preliminary experiments, two amounts of LAB stimulation were tested (50 and 250 μl) which showed no difference in immune modulation (data not shown). After 16 h of incubation, PBMCs were harvested and washed with PBS, and resulting cell pellet was resuspended in 700 μl of RLT buffer and stored at −80°C.

### Quantitative Expression Analysis by Quantitative Real-Time PCR

RNA extraction of the samples was performed using Direct-Zol RNA Miniprep Plus (ZYMO Research). All steps were performed at room temperature and centrifugation at 10,000 × *g* for 30 s. First, an equal volume (700 μl) of ethanol (95–100%) was added to a sample lysed in RLT buffer and mixed thoroughly. The mixture was transferred into a Zymo-Spin™ IIICG Column2 in a collection tube and centrifuged. The column was transferred into a new collection tube and the flow-through was discarded. For DNase treatment, 400 μl RNA Wash Buffer was added to the column and centrifuged. Then, in an RNase-free tube, 5 μl of DNase I (6 U/μl) and 75 μl DNA digestion buffer were added. After incubation at room temperature (20–30°C) for 15 min, 400 μl of Direct-zol™ RNA PreWash5 was added to the column and centrifuged. About 700 μl of RNA Wash Buffer was then added to the column and centrifuged for 1 min to ensure complete removal of the wash buffer. Eventually, the mix was transferred to the column into an RNase-free tube. Finally, 50 μl of DNase/RNase-Free water was added directly to the column matrix and centrifuged, and the RNA was collected in a 1.5-ml Eppendorf tube. The quantity and purity of RNA were assessed using a NanoDrop ND-1000 Spectrophotometer (Thermo Fisher Scientific, MA, USA). The A260:280 ratio was in a range of 2.0–2.2 for all samples, and RNA was resuspended to a final concentration of 1 μg/μl. Total RNAs from each sample were reverse-transcribed and treated with RNAse using the SuperScriptTM III First-Strand Synthesis System using random hexamer primers (Invitrogen™, Life Technologies™, USA) according to the manufacturer's instruction. Generated complementary DNA (cDNA) was stored at −20°C or used directly for amplification at a working dilution of 1:100 (38).

A panel of 26 immune markers, including 18 cytokines: tumor necrosis factor alpha (TNFα), interferon alpha (IFNα), interferon gamma (IFNγ), interleukin (IL)1α, IL6, IL15, IL17, IL18, IL21, IL1β, IL2, IL23, IL8, IL12β, IL10, IL5, IL13, transforming growth factor beta (TGFβ) related to Th1, Th2, Th17, and T regulatory (Treg) responses; two transcription factor genes: nuclear factor kappa-light-chain-enhancer of activated B cells (NFKb)50, NFKb65; six pathogen-recognition receptors: retinoic acid-inducible gene I (RIG-1), toll-like receptor (TLR)2, TLR3, TLR4, TLR9, cluster of differentiation 163 (CD163), and glyceraldehyde 3-phosphate dehydrogenase (GAPDH) as a housekeeping gene, was generated and used to evaluate gene expression using quantitative real-time PCR (Bio-Rad). Primers were either obtained from previous studies or designed using NCBI-Primer BLAST using targeted swine genes (Supplementary Table S1). The primers were validated by melting curve analysis. Quantitative real-time PCR (qPCR) was performed as previously described (38). Briefly, qPCRs were set up for diluted cDNA samples (1:100) in a final volume of 20 μl using iQTM SYBR® Green Supermix (Bio-Rad Laboratories, Hercules, USA) and primers at 1.25 μM concentration. The qPCR was performed in a CFX96TM Real-Time PCR detection system (Bio-Rad) with an initial denaturation step of 3 min at 95°C, followed by 40 cycles of 10 s at 95°C, 20 s at 59°C, and 20 s at 72°C with fluorescence read during extension. The melting curves (Tm) of amplicons were analyzed at 65–95°C with 0.5°C increments for every 5 s. Template controls (NTC) without cDNA template were run in parallel. qPCR for samples and controls (*n* = 5) was run in triplicates. *C*_q_ values were noted for further analysis, and the melting temperature (*T*_m_) of each amplicon was verified for specificity. Analysis to determine relative gene expression was done using the comparative *C*_t_ (Δ*C*_t_) method, where the expression of each gene was normalized to GAPDH as internal gene, and overall fold change of targeted genes against untreated controls was calculated as ΔΔ*C*_t_ (39). The choice of GAPDH as the reference gene was made following an efficiency test evaluation of five different housekeeping genes [actin, 18S, GAPDH, cyclophilin, and peptidylprolyl isomerase A (PPIA)] with the web-based tool RefFinder (40) which, by integrating the major available computational programs (geNorm, NormFinder, BestKeeper, and the comparative Delta-Ct method), ranked GAPDH as the most stable gene among the ones evaluated (data not shown).

### Statistical Analysis

To compare different treatments on PBMCs, one-way ANOVA (Kruskal–Wallis test) followed by Dunn's multiple comparisons test was performed using GraphPad Prism. Statistically significant (*P* < 0.05) gene-expression differences were graphically represented by a separated scatter graph showing individual and mean values. Heat maps showing hierarchical clustering based on one minus Pearson correlation were generated using the software Morpheus (https://software.broadinstitute.org/morpheus).

## Results

### The Irradiation Dose Needed to Inhibit the Replication Is Strain-Dependent in LAB

**Figure 1:**
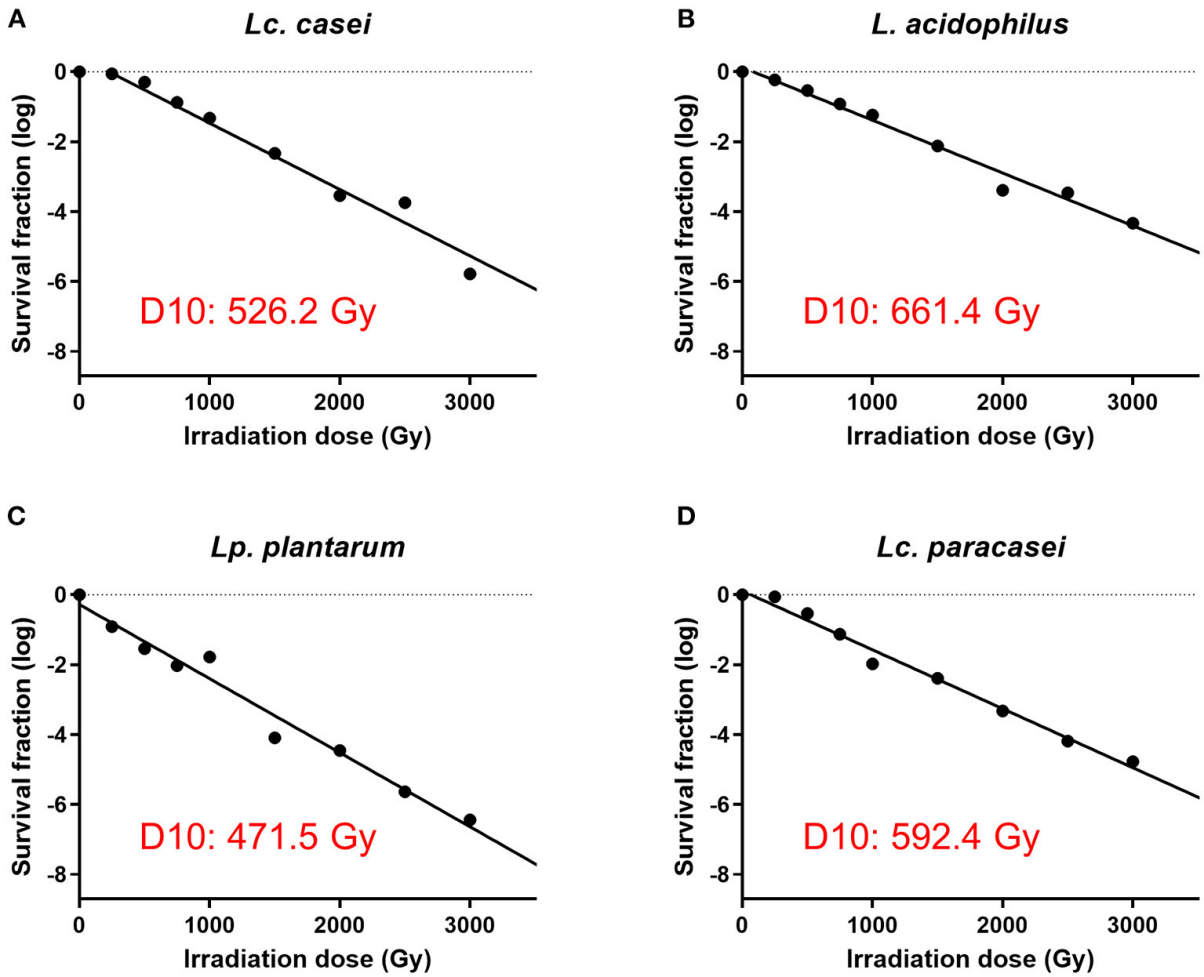
Assessment of surviving fraction at increasing irradiation doses and calculation of the D10 value. The four strains of LAB were irradiated with eight increasing doses of gamma irradiation (250, 500, 750, 1,000, 1,500, 2,000, 2,500, and 3,000 Gy) and the surviving fraction of bacteria was enumerated. D10 value of each strain was calculated using the inverse of the slope of the regression lines (−1/slope) of gamma irradiation dose against survival fraction (log) using GraphPad Prism 9. D10 values are as follows: 526.2 Gy for *Lacticaseibacillus casei* **(A)**, 661.4 Gy for *Lactobacillus acidophilus* **(B)**, 471.5 Gy for *Lactiplantibacillus plantarum* **(C)**, and 592.4 Gy for *Lacticaseibacillus paracasei* **(D)**.

In our first experiment, we determined the irradiation dose that is needed to stop the replication of four strains of LAB, namely, *Lc. casei, L. acidophilus, Lc. paracasei, and Lp. plantarum*. This was done by treating LAB with increasing doses of gamma irradiation and enumerating the surviving fraction of bacteria. The D10 values of each strain were calculated, where the D10 value represents the dose of irradiation needed to lower the concentration of an organism by one log. The results showed (Figure 1 and Supplementary Figure S1) that the D10 values assessed were 526.2, 661.4, 592.4, and 471.5 Gy for *Lc. casei, L. acidophilus, Lc. paracasei*, and *Lp. plantarum*, respectively; thus, at concentrations of 10^9^ cfu/ml, the minimum dose needed for the complete inhibition of replication was estimated as 4,735.8, 5,952. Gy, 5,331.6, and 4,243.5 Gy, which were then rounded up to 5,500, 7,000, 6,000, and 5,000 Gy, respectively (SID; as explained in the M&M). Colony counts confirmed that there was no growth following treatments at SID, 10,000 Gy, or after heat treatment, whereas at 3,000 Gy, all the strains produced colonies.

### Lethal Irradiation Preserves the Membrane Integrity and the Metabolic Activity in LAB

**Figure 2:**
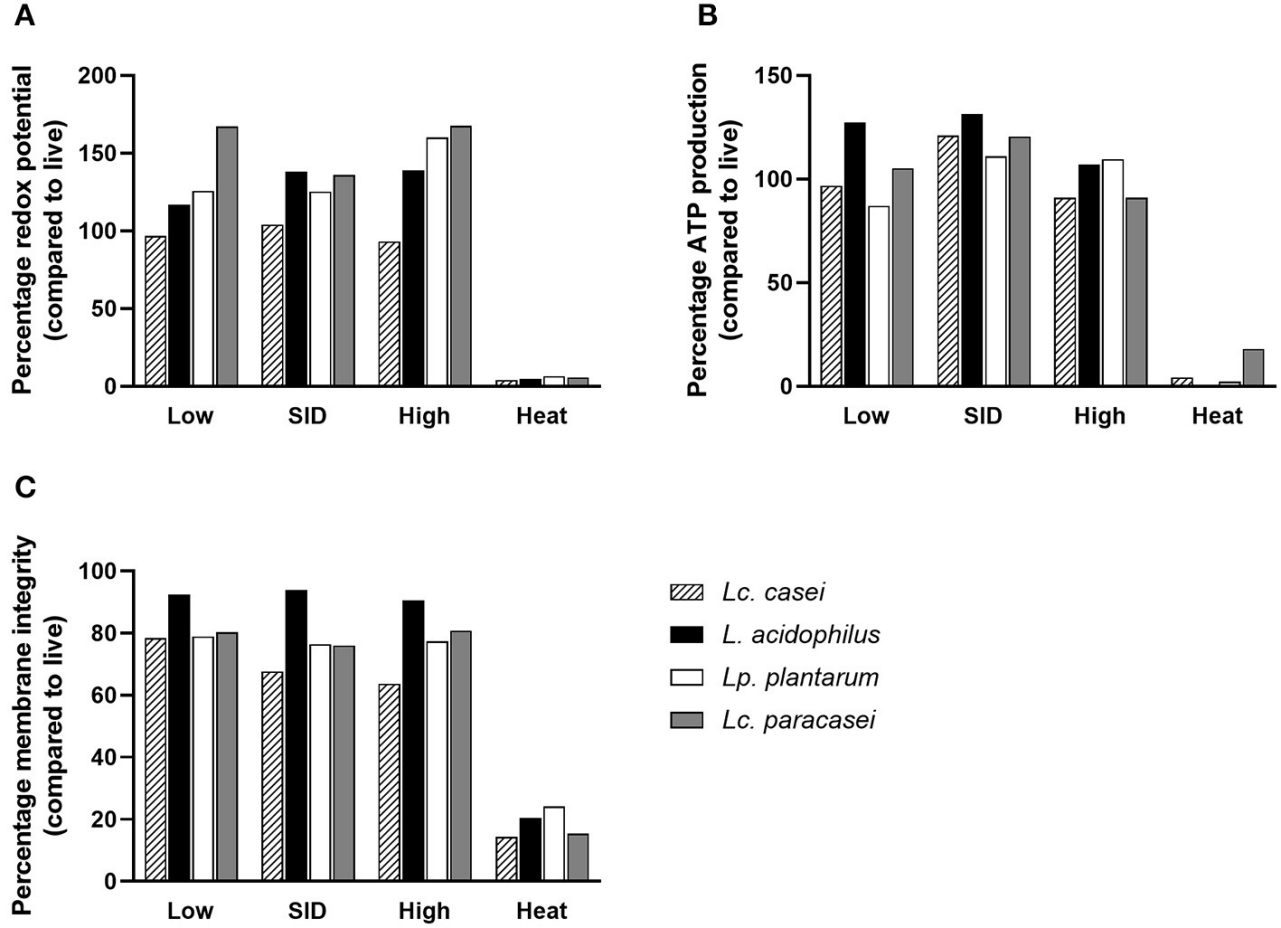
Metabolic activity and membrane integrity. Percentages of redox potential **(A)**, ATP production **(B)**, and membrane integrity **(C)** of irradiated and heat-inactivated *Lacticaseibacillus casei* (pattern-filled), *Lactobacillus acidophilus* (black), *Lactiplantibacillus plantarum* (white), and *Lacticaseibacillus paracasei* (gray) compared to live bacteria are shown. Mean values derived from triplicates are shown in each graph. Irradiation doses: low (3,000 Gy), SID (variable doses according to the strain), high (10,000 Gy); heat treatment: 95°C, 10 min.

Since few reports (30, 41) have stated the ability of gamma-irradiated replication-incompetent bacteria to maintain residual metabolic activity, an experiment was conducted to assess whether the LAB strains in our study were able to preserve metabolic activity in comparison with heat-inactivated and live (as a calibrator) lactic acid bacteria. To further characterize the effect of irradiation on the metabolic activity, we irradiated LAB with three levels of gamma irradiation doses: SID (as stated above, variable doses for each strain), or lower (low; 3,000 Gy), or higher (high; 10,000 Gy) irradiation dose. Different doses of gamma rays depend on the exposure time of the sample to the radiation source (^60^Co). The calculation is based on the *absorbed dose constant*, which is related to the decay energy and time of the radioactive source. For ^60^Co, it is equal to 0.35 mSv/ (GBq h) at 1 m from the source. This allows the calculation of the equivalent dose, which depends, as described, on distance and activity. Therefore, the three doses were delivered by calculating the exposure time based on the current dose rate (on the day that irradiation was performed) (34). To characterize treated LAB, two parameters were measured: metabolic activity (as redox potential and ATP production) and membrane integrity. Results suggest that metabolic activity of irradiated LAB was preserved at all three doses tested (Figure 2), corroborating the findings of other reports. Interestingly, metabolic activity was preserved even at higher doses (10,000 Gy) of irradiation despite delivering a dose nearly that of two times the SID. Surprisingly, we report that the metabolic activity was even higher following irradiation compared to live bacteria in terms of redox potential and ATP production. Conversely, heat inactivation led to less or no metabolic activity post-treatment. In the case of membrane integrity, a parameter that reflects structure preservation, irradiated LAB were able to preserve the membrane integrity although less than the live bacteria (as a percentage), while heat inactivation, as expected, led to a damaged membrane showing minimum membrane integrity.

### Immune Modulation of Swine PBMCs by Live and Treated LAB

#### Immune Modulation by Each Live LAB Has Shared Features but Is Unique to Each Strain

**Figure 3:**
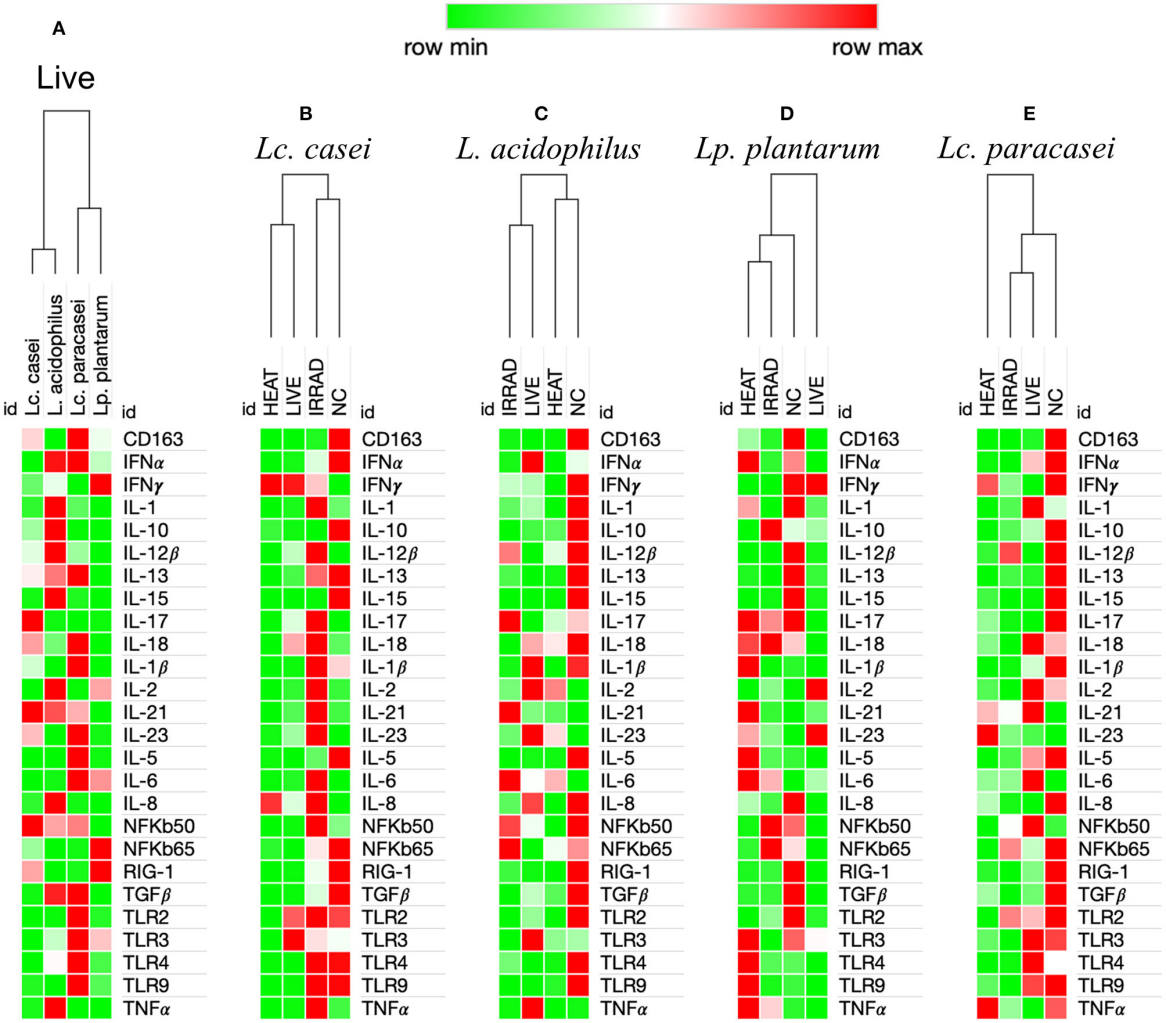
Heat maps analysis of gene expression regulation of 26 immune markers in porcine PBMCs induced by viable and nonviable lactobacilli. Degree of similarity based on one minus Pearson correlation hierarchical clustering in the immune modulation exerted by LAB on the up-(red) or downregulation (green) of 26 immune markers in porcine peripheral blood mononuclear cells. This analysis was performed based upon ΔΔ*C*_t_ differences among the four live lactobacilli strains **(A)**, and based upon Δ*C*_t_ differences among PBMCs unstimulated (NC) and stimulated with live, gamma-irradiated, and heat-treated *Lacticaseibacillus casei* **(B)**, *Lactobacillus acidophilus* **(C)**, *Lactiplantibacillus plantarum* **(D)**, and *Lacticaseibacillus paracasei* **(E)**. Values in the heat map are mapped to colors using the minimum and maximum of each row independently.

Finally, we investigated whether metabolically active non-replicative LAB could resemble their live counterparts in immunomodulatory function. To this end, the expression of 26 immune markers on porcine PBMCs was measured in an *in vitro* end-point assay. We first aimed to identify the degree of immune-modulation similarity among the four strains of LAB that we investigated in their live form. As shown in Figure 3A, according to the hierarchical clustering of immune marker expression heat maps, two disparate similarities were identified. Although being genetically more similar to *Lc. paracasei, Lc. casei* showed similar immune marker expression to *L. acidophilus*, whereas *Lc. paracasei* turned out to be more similar to *Lp. plantarum*.

#### Preservation of Immune Modulation Following Treatment Is More Common With Gamma Irradiation Compared to Heat Treatment

**Figure 4:**
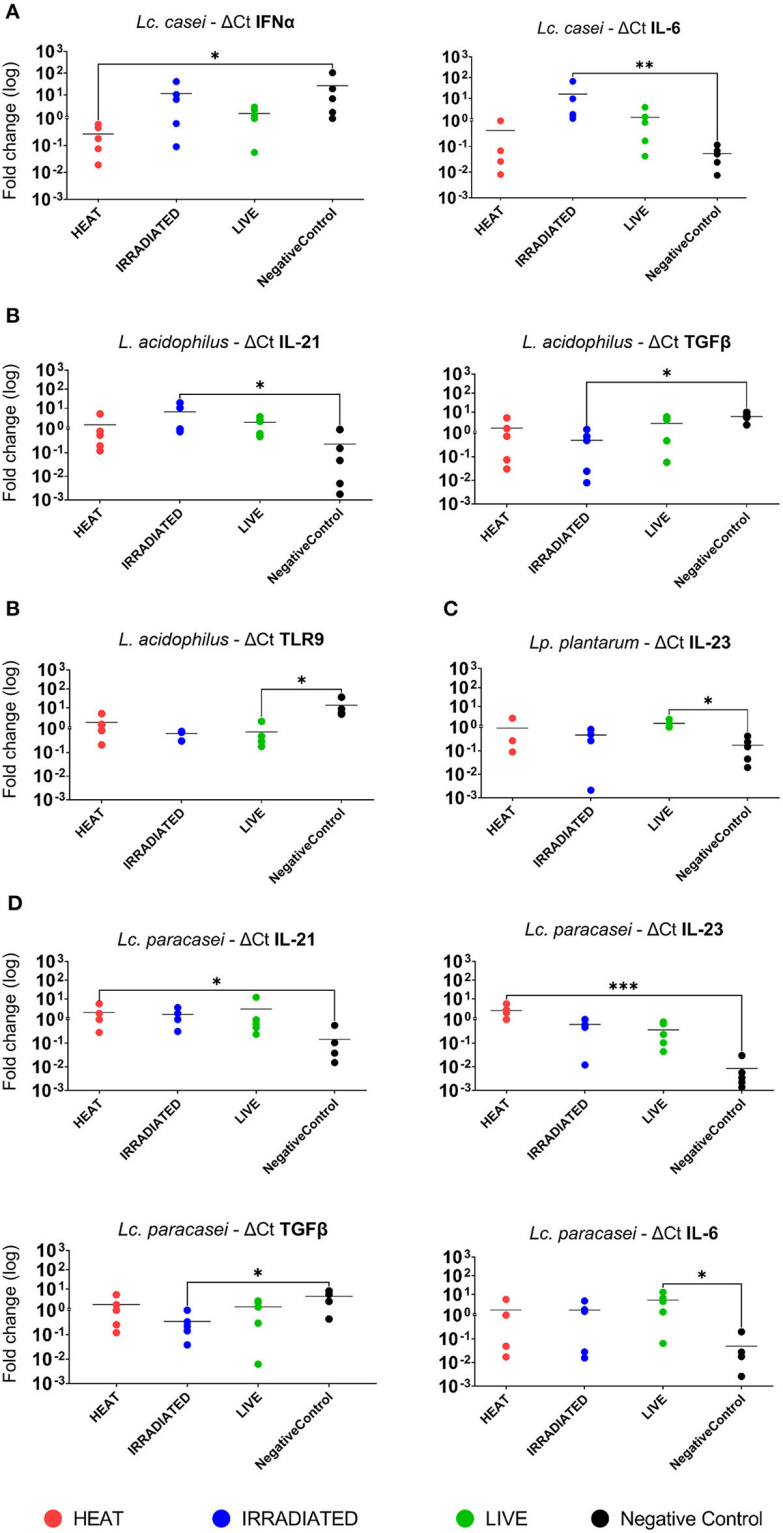
Statistically significant fold change differences in gene expression of immune markers in porcine PBMCs. Logarithmic fold change difference in gene expression comparing individual values (dots) of each animal (*n* = 5) where blood was collected to isolate PBMCs. Here, we report gene expression showing statistical significance highlighted by one-way ANOVA analysis, where * (*P* ≤ 005), ** (*P* ≤ 0.01), and *** (*P* ≤ 0.001) were used to express the degree of significance. Gene expression analysis was performed by comparing unstimulated PBMCs (negative control; black dot) with heat-treated (red dot), gamma-irradiated (blue dot), or live (green dot) *Lacticaseibacillus casei* **(A)**, *Lactobacillus acidophilus* **(B)**, *Lactiplantibacillus plantarum* **(C)**, and *Lacticaseibacillus paracasei* **(D)**.

In a subsequent analysis, we examined how different treatments, such as gamma irradiation or heat inactivation, could alter the ability of each LAB strain in modulating the immune system in comparison with their live state. Unstimulated PBMCs were used as the negative control. According to heat map analysis of global expression of the target immune markers, heat-treated and live *Lc. casei* stimulated gene expression in porcine PBMCs similarly; on the contrary, irradiated *Lc. casei* showed less effect in immune modulation, displaying a profile more similar to the negative control (non-stimulated PBMC) as shown in Figure 3B. When the expression of each target gene was examined individually (Figure 4A), heat-treated *Lc. casei* induced a significant 2.5-fold downregulation of IFNα compared to the negative control, whereas irradiated *Lc. casei* induced a significant 1-fold upregulation of IL-6 compared to the untreated.

Heat maps for *L. acidophilus* (Figure 3C) showed that the irradiated strain could preserve similar immune-modulatory characteristics as the live state, whereas heat treatment led to an immune landscape comparable to the untreated, showing less or no effect. In terms of individual gene expression, irradiated *L. acidophilus* was able to induce a significant 1.5-fold upregulation of IL-21 and a significant one-fold downregulation of TGFβ, whereas live *L. acidophilus* induced a significant one-fold downregulation of TLR9 expression (Figure 4B).

Gene expression analysis of the different states of *Lp. plantarum* was performed with the same method, showing similarity in terms of overall immune marker expression between the two treatments (irradiation and heat); furthermore, both treated versions of *Lp. plantarum* showed similar overall gene expressions to the untreated cells. Not surprisingly, instead, live *Lp. plantarum* seemed to induce a very different stimulation of gene expression compared to treated strains and to the untreated samples (Figure 3D). The only significant difference in gene expression was observed for live *Lp. plantarum*, which was able to induce a 1-fold upregulation of IL-23 compared to untreated PBMCs (Figure 4C).

Finally, the same type of analysis was performed for *Lc. paracasei*, showing again that irradiated treatment induced an overall gene expression of the targeted immune markers comparable to the strain's live state. In general, both live and irradiated *Lc. paracasei* were unable to stimulate the whole pool of markers observed for the untreated cells. In contrast, heat-treated *Lc. paracasei* induced a very different stimulation of gene expression compared to the irradiated and live *Lc. paracasei* and compared to the untreated samples (Figure 3E). Heat-treated *Lc. paracasei* was able to induce a significant one-fold and a 2.5-fold upregulations of IL-21 and IL-23, respectively, compared to the untreated samples; instead, live *Lc. paracasei* was able to induce a significant two-fold upregulation of IL-6 whereas the irradiated version induced a significant one-fold downregulation of TGFβ (Figure 4D).

**Figure 5:**
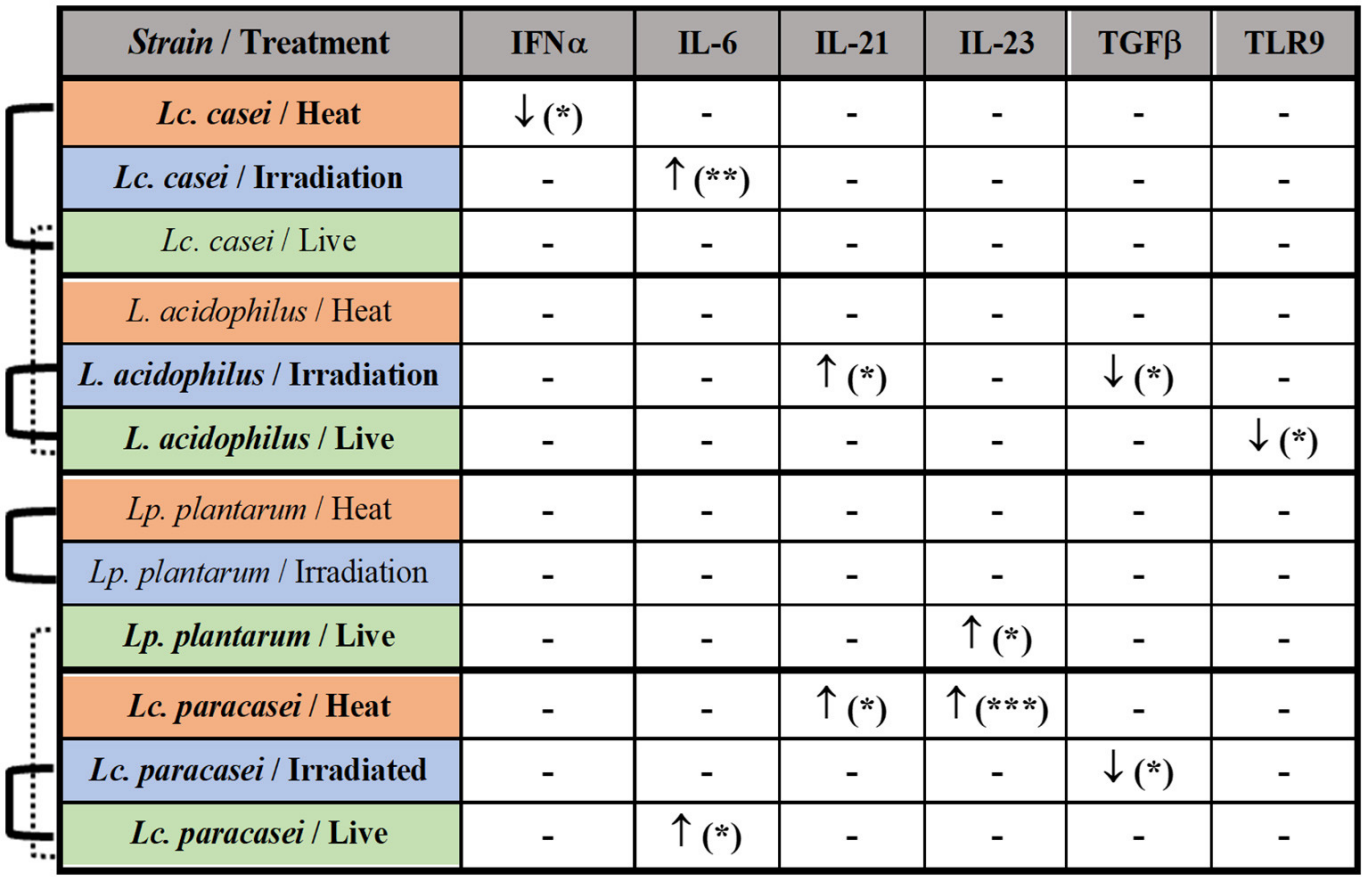
Summary of the immune modulation exerted by immunobiotic and paraprobiotic LAB strains. LAB strains and respective treatments are listed in the first column. Strains highlighted in bold were able to induce a statistically significant difference in the up- (↑) or downregulation (↓) of the immune markers listed in the first row. Similarities in overall gene expression modulation, observed in heat maps hierarchical clustering, are indicated by connection lines on the left side of the table. Solid lines show similarity among different states of the same strain whereas the dashed line shows a similarity among live strains of different genera. * *P* ≤ 0.05, ** *P* ≤ 0.01 and *** *P* ≤ 0.001 were used to express the degree of significance.

Taken together these results, as shown in the summary (Figure 5), the most affected immune markers by the stimulation of LAB are four pro-inflammatory cytokines (IFNα, IL-6, IL-21, and IL-23), one anti-inflammatory cytokine (TGFβ), and a pathogen recognition receptor (TLR9); two of four LAB strains evaluated in this study were able to maintain an overall gene expression similar to their live state (showed also in Supplementary Figures S2–S5) after gamma irradiation; heat treatment and irradiation of *Lc. casei* led to an opposite regulation of key pro-inflammatory cytokines, such as downregulation of IFNα for heat-treated *Lc. casei* and upregulation of IL-6 for irradiated *Lc. casei*; gamma irradiation of *L. acidophilus* and *Lc. paracasei* led to a downregulation of TGFβ.

## Discussion

This study aimed at investigating the immune-modulatory effects of gamma irradiated, non-replicative, yet metabolically active LAB. Our study found that irradiated LAB could preserve the immune-modulatory blueprint of their live state more than heat-inactivated bacteria. The end-point gene expression of a broad array of immune markers expressed by porcine PBMCs, evaluated by one minus Pearson correlation hierarchical clustering upon stimulation with irradiated, heat-killed, or live strains of LAB, provides this evidence. However, being metabolically active as their live form does not necessarily implicate similar immune responses when a larger immune landscape is evaluated.

A total of two out of four strains (*L. acidophilus and Lc. paracasei*) were able to induce an overall gene expression similar to that of live LAB following irradiation inactivation. On the other hand, we also observed other outputs, such as a higher degree of similarity between live and heat-killed (*Lc. casei*) LAB, or where both treatments led to a similar change in gene regulation differently from the viable state (*Lp. plantarum*). Similar outputs were also observed in other studies (42, 43), but the type of comparison on immune modulation was either limited to one single strain of LAB, whenever the aim of the study was to compare differences induced by the type of treatment, or to one single type of treatment (heat inactivation) when assessing strain-dependent effects. In addition, key factors, such as selection of LAB strain, dose, duration of the stimulation (incubation), and the number of immune markers evaluated often vary among different studies, influencing the outcome of the research. This heterogeneous landscape in terms of variation contributes to making literature on this topic rather contradictory and confusing. Considering these aspects, we decided to adopt a broad approach, by evaluating four different strains of LAB, with two different methods of inactivation, assessing the metabolic activity and the immune modulation based on the expression of 26 immune markers.

We observed an interesting, varied mosaic of statistically significant differences in the regulation of some of the key genes involved in immune modulation. This variability can be justified by the strain-dependent response to inactivation methods among LAB, as for bacteria in general, documented in previous studies, especially on radiation resistance (28), structure composition (31), immune stimulation (25), and adhesion abilities (23), just to name a few. For example, IFNα, mostly involved in antiviral activity (44), was downregulated by heat-killed *Lc. casei*, whereas IL-6, which is a pivotal pro-inflammatory cytokine responsible for regulating the immune response, playing a key role in stimulating B-cell differentiation (45, 46), was upregulated by live *Lc. paracasei* and gamma-irradiated *Lc. casei*. IL-21, another pro-inflammatory cytokine, which plays a role in Th17 development [whose modulation seems to play an important role in adjuvant development (47)], as well as in the proliferation of T cells and differentiation of B cells into memory cells (48), was found to be upregulated in both gamma-irradiated *L. acidophilus* and in heat-treated *Lc. paracasei*. Live *Lp. plantarum* and heat-treated *Lc. paracasei* were able to upregulate IL-23, which is one of the major effector molecules for Th17 maturation (49). Both gamma-irradiated treated *L. acidophilus* and *Lc. paracasei* induced downregulation of the immunomodulatory TGFβ, which is mainly involved in suppressing T- and B-cell action (50) while activating Treg cell responses (24). Finally, live *L. acidophilus* induced a downregulation of toll-like receptor 9 (TLR9), which has been proven to be essential for probiotics to exert an anti-inflammatory effect (51). The variation in the D10 values and therefore in the dose required to stop the replication depends on the bacterial titer and on the specific strain, as seen in the literature (23, 31). Indeed, the individual animal variation could have masked a better analysis of data in a group of five animals.

Interestingly, we observed that strains, which are more resistant to gamma irradiation and would therefore require a higher dose to halt replication (as we showed in the D10 values of assessment), are more likely to retain characteristics of the live state. This can be due to the diverse cell surface structure of the different strains, as it is for instance between *L. acidophilus* (coated with S-layer) and *Lp. plantarum* (un-coated) (52). In fact, the presence of S-layer proteins on the bacterial surface has been shown to augment the resistance to gamma irradiation (53), and we hypothesize that representing the outer layer of protection, the S-layer could be able to absorb most of the radiation effects preserving more efficiently the cell wall components responsible of immune modulation.

The major advantage of irradiation is that strains are inactivated by irradiation, are unable to reproduce, and are considered as safe. The inability to replicate impairs the possibility of mutations. For this reason, we decided to add an extra dose of irradiation on top of the minimum inhibitory dose making sure that these bacteria are completely replication-incompetent. For instance, several pathogenic bacteria, such as *Salmonella* or *Staphylococcus aureus*, have been proposed as safer vaccines when irradiated than inactivated with other technologies (54, 55). Ionizing radiation technology, such as gamma or E-beam irradiation, is able to damage the nucleic acid of the organism, by inducing polymerization of the DNA and breaking molecular bonds (56, 57), without affecting cell functions or the main components of the cells (58) which are destroyed in other inactivation methods, such as heat treatment. Irradiated non-replicative lactic acid bacteria share the key features with both live and heat-treated probiotics. On one hand, through this technology, LAB can retain cellular membrane integrity and metabolic activity (in some studies up to 9 days post-irradiation) similar to live strains. In addition, in some studies, cells were even able to maintain oxidative function and protein synthesis. On the other hand, it ensures a high level of safety by making pathogens unable to replicate, as it also occurs when inactivated through conventional practices (i.e., heat treatment, chemical inactivation). The main disadvantage of this technology is surely represented by the safety concern of hosting a radioactive isotope (i.e., cobalt-60) in the facility (55). To ensure radio- and cryo-protection to the cell wall of the LAB used for this study, we added trehalose to our samples prior to irradiation. Our results show that at all three doses of gamma irradiation (low, SID, and high), the metabolic activity was preserved, as it was consistent with other studies (30, 41). Surprisingly, in most of the cases, metabolic activity after irradiation was even higher than the metabolic activity of live bacteria in terms of redox potential and ATP production. Based on the previous studies, where some bacteria have shown the ability to maintain transcription and translation processes for a limited time following gamma irradiation (59), we can hypothesize that the augmented metabolic activity of irradiated LAB can be seen as an effort performed by the cell to counteract the damages caused by ionizing radiation. In fact, according to Acharya et al. (60) and Abomohra et al. (61), the increased metabolic activity indeed reflects a compensatory response in cells damaged by gamma irradiation in an effort to survive. One direct consequence of an upregulated metabolism is oxidative stress, and irradiation leads to a significant increase in reactive oxygen species/reactive nitrogen species (ROS/RNS) levels. Furthermore, gamma irradiation induces an increased extracellular release of ATP, which stimulates the production of ROS *via* purinergic signaling, leading to the promotion of intracellular antioxidant production, such as pigments and proteins, in response to oxidative stress. We also found that the membrane structural integrity of irradiated LAB is similar to live cells, but different from heat-treated cells. The reason behind, as previously described by other reports (31, 62), is that γ-rays have no impact on membrane lipid profile, nor on peroxidation events, indicating the plausible preservation of the membrane-bound proteins.

A positive correlation between the preservation of metabolic activity after gamma irradiation and immune stimulation capacity was highlighted in some studies, showing better immunogenicity exerted by irradiated compared to heat-inactivated bacteria (41, 63, 64). In addition, studies have demonstrated that compounds of bacterial cells (e.g., teichoic acid, cell wall polysaccharides, and exopolysaccharides) are plausibly the main causative agents for the pro- or anti-inflammatory effects exerted by these microorganisms (21, 26) and that the exposure to high temperatures due to the heat treatment induces denaturation and coagulation of these proteins (58). This does not imply that an immune-modulatory activity of heat-treated strains is not expected. On the contrary, studies have demonstrated that immune modulation can be even more pronounced (25), but that would differ from the viable state.

In this study, we decided to analyze the gene expression of a set of 26 immune markers at a precise time point (16 h after co-incubation), which has been selected also in other studies (65, 66), representing a “snap-shot” of the immune-modulatory action, known to be a dynamic process. This study represents the first screening of the quest for a gamma-irradiated LAB product, which can potentially be incorporated as a vaccine adjuvant or immune therapeutic in the future. The intrinsic immune-modulatory capacity of the LAB combined with the safety conferred by irradiation may generate a product that can be broadly applied to the animal health field, beyond the usage as immune modulators. However, careful strain selection, kinetic gene expression analyses, and additional *in vitro, in vivo* as well as protein arrays are encouraged to further evaluate the application of irradiated LAB as vaccine adjuvants or immune-modulatory therapeutics.

## Data Availability Statement

The original contributions presented in the study are included in the article/Supplementary Material, further inquiries can be directed to the corresponding author.

## Ethics Statement

The animal study was reviewed and approved by University of Veterinary Medicine Vienna's Ethics and Animal Welfare Committee and Austrian Ministry of Research and Science's Advisory Committee for Animal Experiments (BMBWF-68.205/0192-V3b/2018).

## Author Contributions

VW, KD, LP, and GC: contributed to the conception and design of the study. LP, JB, VW, and RK: performed experiments and analyzed the data. DV: provided veterinary support and provided swine blood. LP and JB: wrote the first draft of the manuscript. All authors contributed to manuscript revision, read, and approved the submitted version.

## Conflict of Interest

The authors declare that the research was conducted in the absence of any commercial or financial relationships that could be construed as a potential conflict of interest.

## Publisher's Note

All claims expressed in this article are solely those of the authors and do not necessarily represent those of their affiliated organizations, or those of the publisher, the editors and the reviewers. Any product that may be evaluated in this article, or claim that may be made by its manufacturer, is not guaranteed or endorsed by the publisher.
