# Supplementary material for: Irradiated Non-replicative Lactic Acid Bacteria Preserve Metabolic Activity While Exhibiting Diverse Immune Modulation
Source: Front Vet Sci. 2022 May 18;9:859124. doi: 10.3389/fvets.2022.859124 (PMC9158532; doi:10.3389/fvets.2022.859124)
Supplement: Supplementary file 1 [file Data_Sheet_1.docx]

**Supplementary Figure 1.** Assessment of surviving fraction at increasing irradiation doses and calculation of the D10 value. The four strains of LAB were irradiated with eight increasing doses of gamma irradiation (250 Gy, 500 Gy, 750 Gy, 1,000 Gy, 1,500 Gy, 2,000 Gy, 2,500 Gy and 3,000 Gy) and the surviving fraction of bacteria was enumerated. D10 value of each strain was calculated using the inverse of the slope of the regression lines (-1/slope) of gamma irradiation dose against survival fraction (log) using GraphPad Prism 9.

**Supplementary Figure 2**. Immune markers gene expression of *Lc. casei* in porcine PBMCs. Logarithmic fold-change difference in gene expression comparing individual values (dots) of each animal (n=5) where blood was collected from to isolate PBMCs. Here we report gene expression showing statistical significance highlighted by One-way ANOVA analysis, where * (P ≤ 0.05), ** (P ≤ 0.01) and *** (P ≤ 0.001) were used to express the degree of significance.

**Supplementary Figure 3**. Immune markers gene expression of *L. acidophilus* in porcine PBMCs. Logarithmic fold-change difference in gene expression comparing individual values (dots) of each animal (n=5) where blood was collected from to isolate PBMCs. Here we report gene expression showing statistical significance highlighted by One-way ANOVA analysis, where * (P ≤ 0.05), ** (P ≤ 0.01) and *** (P ≤ 0.001) were used to express the degree of significance.

**Supplementary Figure 4**. Immune markers gene expression of *Lc. paracasei* in porcine PBMCs. Logarithmic fold-change difference in gene expression comparing individual values (dots) of each animal (n=5) where blood was collected from to isolate PBMCs. Here we report gene expression showing statistical significance highlighted by One-way ANOVA analysis, where * (P ≤ 0.05), ** (P ≤ 0.01) and *** (P ≤ 0.001) were used to express the degree of significance.

**Supplementary Figure 5**. Immune markers gene expression of *Lp. plantarum* in porcine PBMCs. Logarithmic fold-change difference in gene expression comparing individual values (dots) of each animal (n=5) where blood was collected from to isolate PBMCs. Here we report gene expression showing statistical significance highlighted by One-way ANOVA analysis, where * (P ≤ 0.05), ** (P ≤ 0.01) and *** (P ≤ 0.001) were used to express the degree of significance.
