# Supplementary figures and images for: Irradiated Non-replicative Lactic Acid Bacteria Preserve Metabolic Activity While Exhibiting Diverse Immune Modulation
Source: Front Vet Sci. 2022 May 18;9:859124. doi: 10.3389/fvets.2022.859124 (PMC9158532; doi:10.3389/fvets.2022.859124)

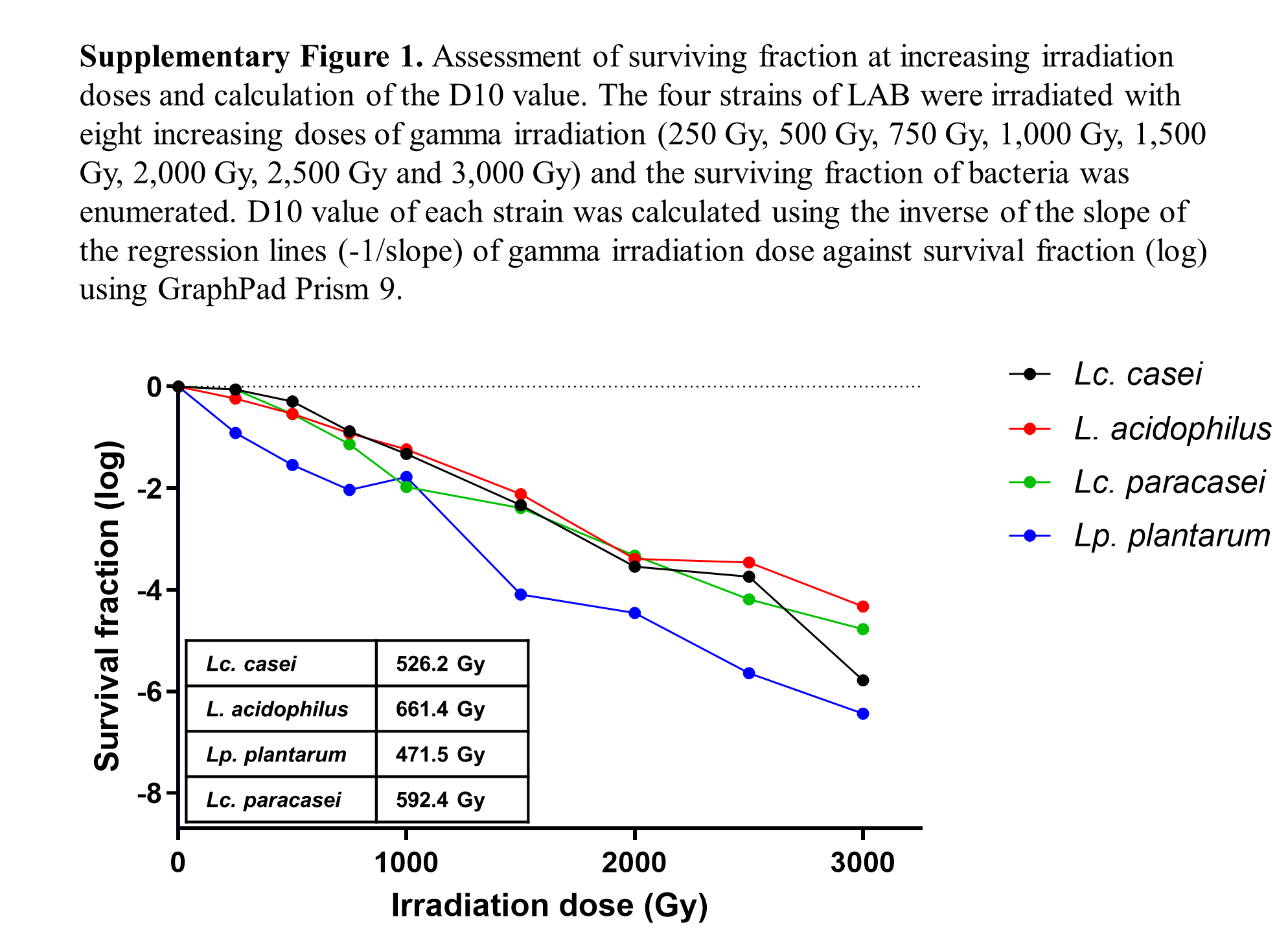

Supplement: Supplementary file 2 [file Image_1.tif]

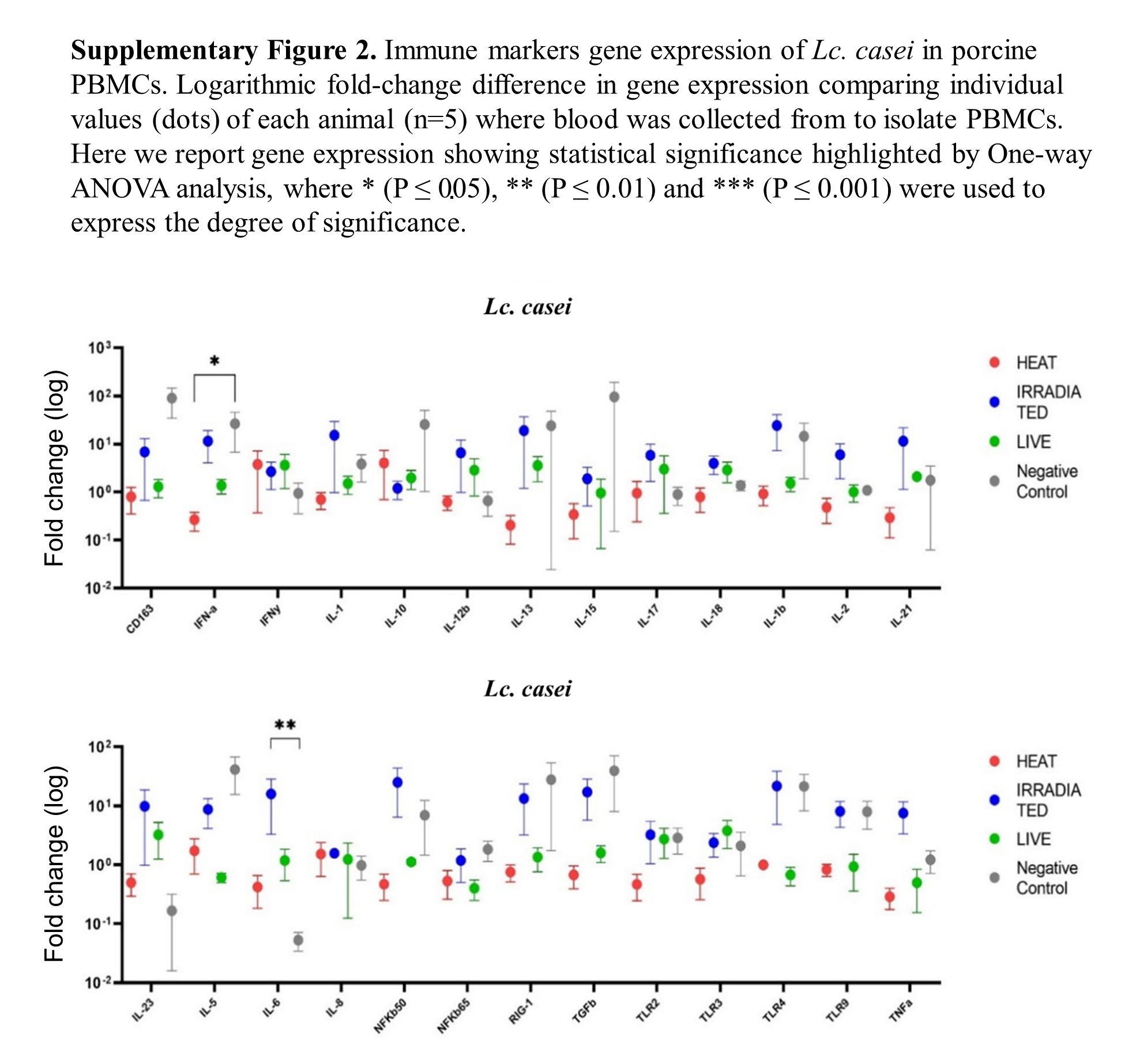

Supplement: Supplementary file 3 [file Image_2.tif]

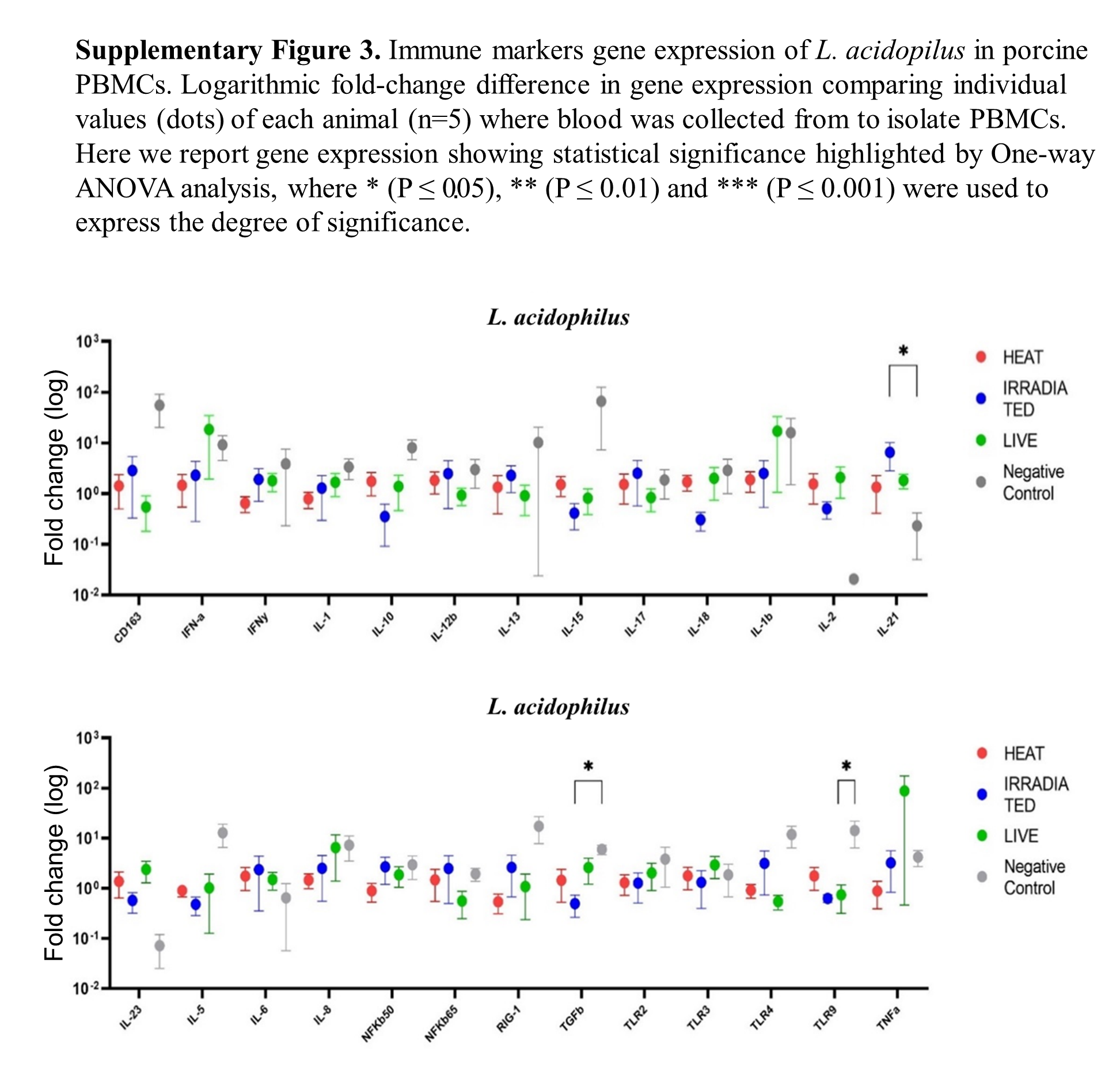

Supplement: Supplementary file 4 [file Image_3.tif]

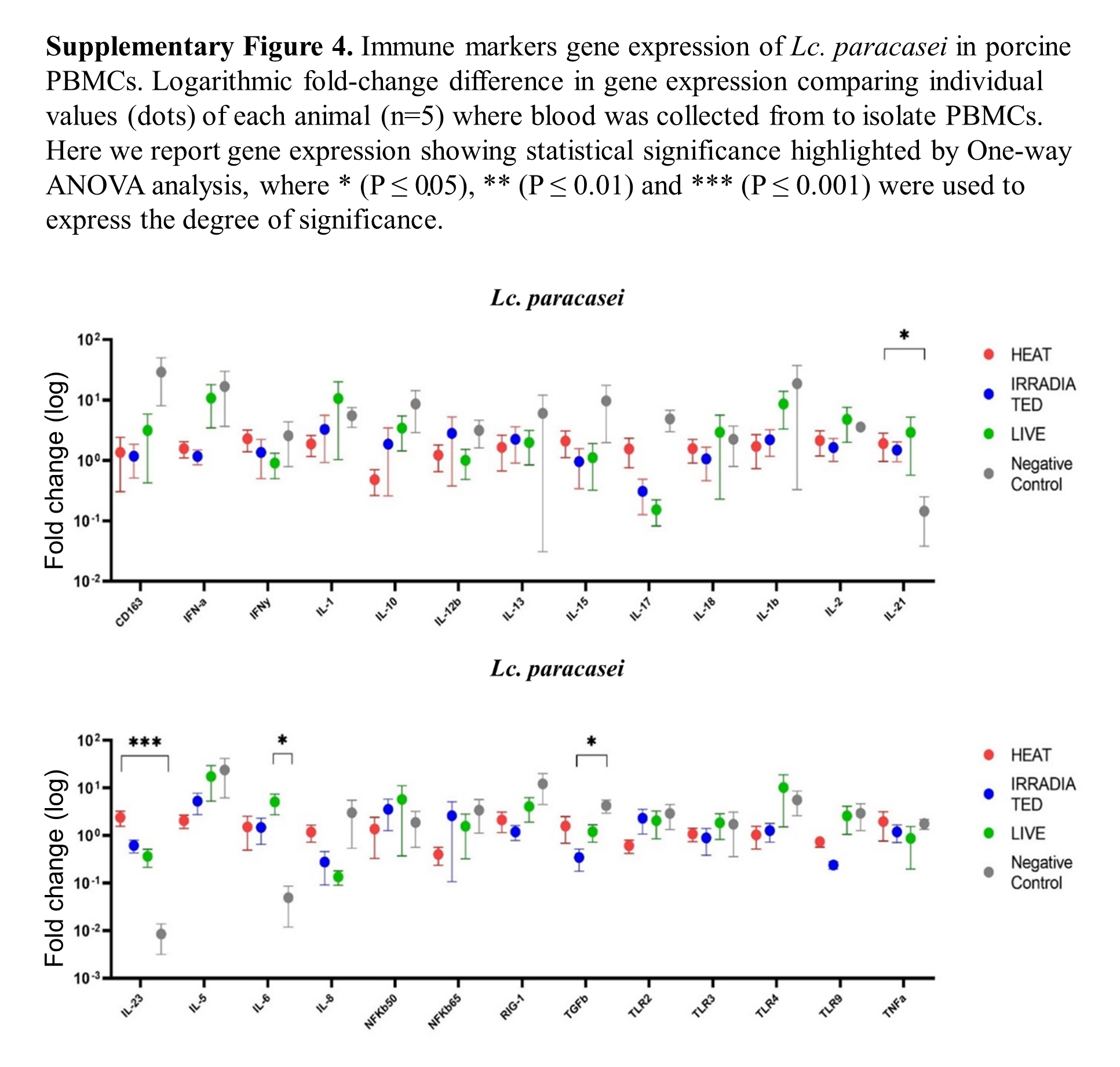

Supplement: Supplementary file 5 [file Image_4.tif]

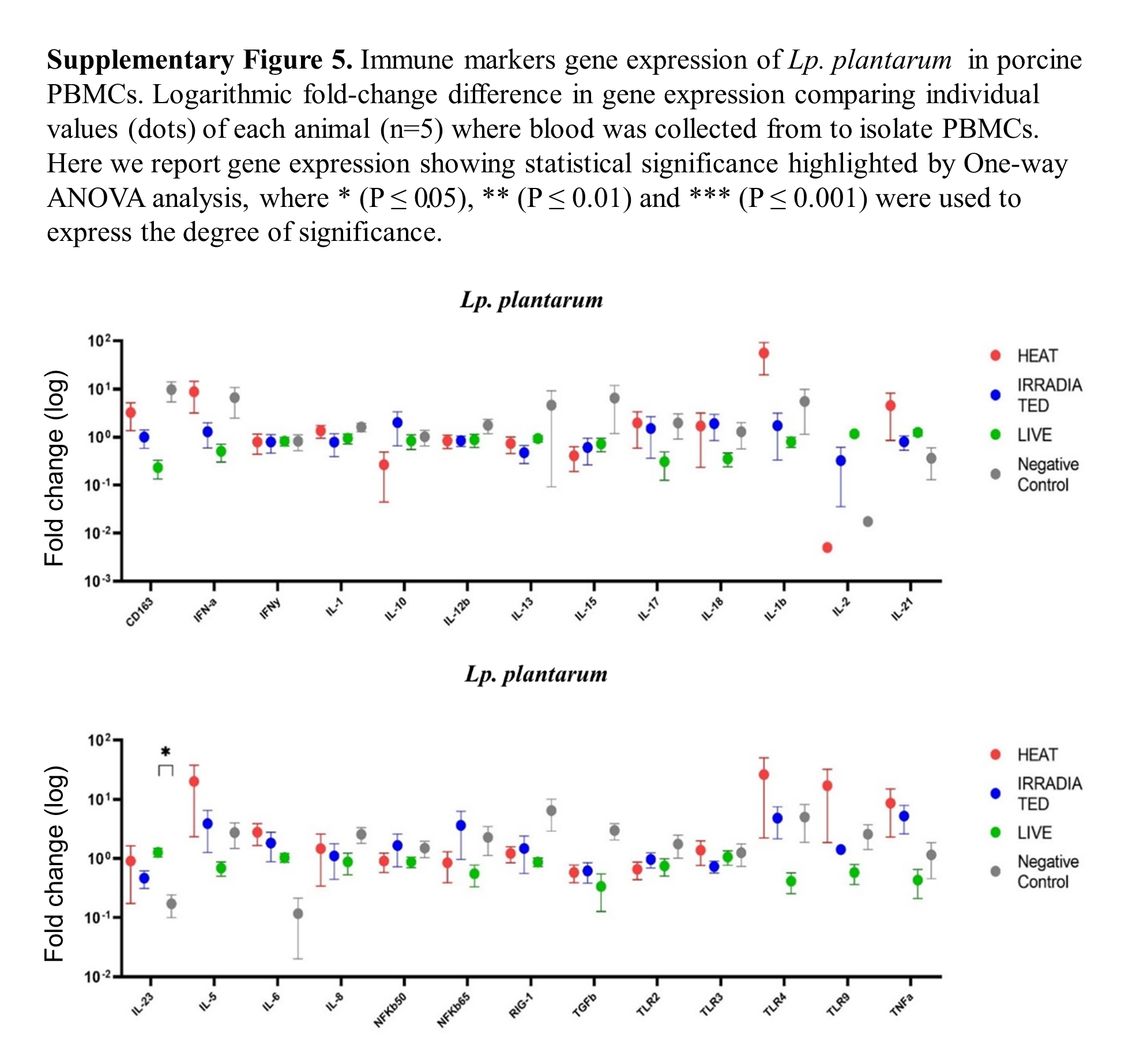

Supplement: Supplementary file 6 [file Image_5.tif]
